# Current Video Gaming Is Associated with Visual–Psychomotor Efficiency and Visuomotor Coordination in Adolescents

**DOI:** 10.3390/brainsci16101140

**Published:** 2026-10-21

**Authors:** Grzegorz Załęski, Krzysztof Horoszkiewicz, Marta Kopańska

**Affiliations:** 1 Faculty of Social Sciences and Computer Science, Academy WSB-NLU, 17 Grunwaldzka Street, 33-300 Nowy Sącz, Poland; gzaleski@wsb-nlu.edu.pl (G.Z.); kontakt@horeszko.eu (K.H.); 2 Department of Medical Communication and Professional Competency Development, Faculty of Medicine, Collegium Medicum, University of Rzeszów, 35-310 Rzeszów, Poland

**Keywords:** adolescents, video gaming, psychomotor performance, visual attention, visuomotor coordination, cognitive performance

## Abstract

**Highlights:**

**What are the main findings?**

- Current video gaming was associated with higher psychomotor throughput and faster visual-search performance in adolescents.
- The observed associations were selective rather than indicative of a generalized cognitive advantage.

**What are the implications of the main findings?**

- Current gaming status may be associated with specific aspects of visual–psychomotor functioning, particularly tasks requiring rapid visuomotor responses.
- The cross-sectional findings do not establish causality, emphasizing the need for longitudinal research to clarify the nature of these associations.

**Abstract:**

Background/Objectives: Video gaming places repeated demands on rapid visual information processing, response selection, and visuomotor coordination. However, it remains unclear whether current gaming in adolescence is associated with broadly enhanced cognitive performance or with selective differences in visual–psychomotor functioning. This study examined associations between current video gaming and visual–psychomotor efficiency, visuomotor coordination, visual search, perceptual performance, and sequential–executive processing in adolescents. Methods: This exploratory secondary cross-sectional reanalysis used an existing dataset of 120 adolescents aged 14–16 years (60 girls, 60 boys), including 92 current and 28 non-current players. Five principal outcomes were analysed using age- and sex-adjusted regression, HC3 standard errors, and Benjamini–Hochberg correction. Sensitivity analyses examined girls, school membership, and all 121 source records. Results: Current gaming was associated with higher PiM (B = 6.92, 95% CI [1.24, 12.59], *q* = 0.043) and shorter Number Localization time (9.6%, *q* = 0.042) in the principal models. PiM estimates remained positive across school and sample sensitivity analyses, although not every leave-one-school-out model met the FDR threshold. Inference for visual-search time was more sensitive to analytical specification. The component analyses described overlapping measures underlying PiM. Intensity, tenure, and genre analyses were inconclusive after correction. Conclusions: Current gaming was associated most consistently with greater psychomotor throughput. Visual-search estimates generally favoured current players but were less stable across specifications. These exploratory cross-sectional findings do not establish a causal benefit, a general cognitive advantage, or an optimal gaming dose or genre.

## 1. Introduction

Video gaming represents one of the most widespread forms of digital activity among children and adolescents, and its relevance has extended beyond purely recreational use [1]. Adolescence has attracted particular research interest as a developmental period characterized by substantial changes in attentional functioning, cognitive control, and the organization of goal-directed behavior [2]. Studies conducted in adolescent samples have suggested that video gaming may be associated with differences in attentional efficiency, information-processing speed, and performance on tasks requiring the integration of perceptual input and motor responses [3,4,5]. Recent large-sample studies have further indicated that associations between gaming and cognitive functioning are not uniform and may depend both on patterns of game use and on the specific cognitive domain under examination [4,6]. This has supported a shift away from a dichotomous view of gaming as either uniformly beneficial or harmful toward identifying the specific cognitive and psychomotor domains associated with different patterns of gaming activity.

From a cognitive–psychological perspective, video games, particularly those characterized by high temporal demands and rapidly changing task conditions, engage several interrelated processes. Players are required to select relevant visual information from competing stimuli, maintain attention on goal-relevant elements, rapidly process changes in the task environment, select an appropriate response, and execute that response with sufficient precision. Video games therefore provide an environment requiring repeated integration across successive processing stages, including stimulus search, information selection, response selection, and motor execution [7,8]. A recent meta-analysis by Zhao et al., comprising 133 studies, identified small but statistically significant associations between video gaming and several cognitive domains, including visual attention and cognitive control [9]. Studies using behavioral and eye-tracking indices of attention have likewise suggested that action-video-game experience may be associated with more efficient visual selection and different patterns of distractor control [3]. With regard to motor performance, recent findings have also demonstrated shorter visual reaction times and better hand–eye coordination among regular players [10]. Taken together, these findings supported the analysis of visual-processing speed, attention, and visuomotor performance as related but distinct components of functioning.

Previous research has suggested that differences between players and non-players are particularly apparent in tasks placing strong demands on speeded processing and visuomotor performance. Argilés et al., studying adolescents aged 15–16 years, found that regular action video game players completed the horizontal condition of the Developmental Eye Movement Test and the King–Devick Test more quickly, indicating greater efficiency in sequential visual search and visual information-processing speed [5]. In the study by Cui et al., action-video-game experience was likewise associated with better performance on a selective visual-attention task, with response time, response accuracy, and eye-tracking parameters examined simultaneously [3]. Szabo et al. in turn, reported shorter bilateral visual reaction times, faster target acquisition, and more favorable selected indices of hand–eye coordination among regular League of Legends players [10]. Broader support for associations between gaming and selected cognitive abilities was provided by the meta-analysis of Zhao et al., which identified small but significant effects for visual attention, spatial ability, and cognitive control [9]. These findings were also consistent with earlier observations of psychomotor and attentional performance among video game players [11,12]. However, the more recent evidence primarily emphasized the need to examine specific components of task performance rather than assume a generalized cognitive advantage among players.

The current literature nevertheless remains inconclusive [13,14,15]. Reported effects have varied as a function of gaming experience, gaming intensity, game type, participant age, and the characteristics of the tasks used. For example, Özçetin et al. found that adolescents with more intensive gaming experience responded more quickly but also made more errors, suggesting a possible speed–accuracy trade-off [16]. A simple dose–response relationship, whereby greater time spent gaming would correspond proportionally to better performance, has likewise not been consistently observed [4,12]. Findings have also depended on game type and game mechanics, while the cross-sectional nature of a substantial proportion of the literature has precluded conclusions as to whether observed group differences reflect gaming-related experience, pre-existing individual differences that increase the likelihood of engaging in gaming, or a combination of both mechanisms. Consistent with this heterogeneity, developmental and large-sample studies have reported positive associations for selected outcomes, nonlinear or exposure-dependent patterns, and null or potentially unfavorable associations for broader cognitive or sustained-attention measures [17,18,19,20,21].

Recent findings have further suggested that searching for a single, generalized effect of gaming may oversimplify the relationship between video gaming and human functioning. Szabo et al. reported player advantages across several indices of reaction time and coordination, but not across all psychomotor parameters examined [10]. Neuroimaging findings have similarly pointed to heterogeneous rather than uniform correlates of gaming. Nagata et al., analyzing data from 5686 adolescents participating in the Adolescent Brain Cognitive Development (ABCD) Study, identified associations between different gaming behaviors and distinct patterns of cortical morphology, with the nature of these associations varying according to characteristics such as gaming duration and type. These findings therefore did not support a simple assumption of either globally beneficial or globally detrimental associations between gaming and cognitive functioning [6].

Against this background, an important unresolved question was whether current video gaming in adolescence was associated with a broad performance advantage encompassing both basic perceptual–motor processes and more complex executive functions, or rather with a selective profile of differences concentrated in visual-processing speed, visual-search efficiency, and visuomotor coordination.

The present study reanalyses the dataset previously reported by Załęski and Warchoł [12]. Its added contribution is to assess which associations remain after adjustment for age and sex and correction for multiple testing, and how their interpretation changes when sex imbalance, school membership, and sample composition are considered. The analysis therefore refines the inference from an existing dataset rather than providing an independent replication.

## 2. Materials and Methods

### 2.1. Study Design and Participants

This was a secondary cross-sectional reanalysis of an existing school-based dataset; no new participants were recruited and no additional measurements were collected. The earlier report by Załęski and Warchoł [12] included 121 adolescents and examined overlapping outcomes, including PiM, coordination measures, Number Localization, Line Test, and CCTT, in relation to gaming frequency or genre. Its analyses included unadjusted Kruskal–Wallis tests, Dunn–Bonferroni comparisons, and Mann–Whitney tests. The present study re-estimates associations using covariate adjustment, explicit multiple-testing families, and sensitivity analyses. The reanalysis was not preregistered; all analyses are exploratory, and “principal” identifies the reporting priority rather than prospective confirmation.

The final sample comprised 120 adolescents aged 14–16 years (M = 14.99, SD = 0.67), including 60 girls and 60 boys. Ninety-two participants (76.7%) reported currently playing video games, whereas 28 (23.3%) reported that they did not currently play. The largest age group consisted of 15-year-old students (n = 67, 55.8%), followed by 14-year-olds (n = 27, 22.5%) and 16-year-olds (n = 26, 21.7%). Participants were recruited from six public secondary schools in southern Poland (Małopolskie and Podkarpackie voivodeships)—three general secondary schools, two technical schools, and one vocational school—after approximately 20 schools had been invited to participate. The source study included 121 first-grade students; the present reanalysis used 120 after the data-verification exclusion described below [12]. Detailed description Table 1.

Schools were approached by telephone and email. Recruitment involved available first-year students during school hours with teachers’ involvement and voluntary participation. The number of individually invited students and the number declining participation were not retained, so a student participation rate cannot be calculated. Eligibility for the present analysis required membership of the source cohort, age 14–16 years, and complete principal-outcome data. The sample size was determined by the available source dataset rather than a new recruitment target.

One source record was excluded from the principal analysis because its simple-coordination measurements were internally inconsistent: a mean response time of 1.43 s was recorded alongside 57 correct responses in a 60 s task. Their product (81.51 s) exceeded the available task duration. The final analytical sample comprised 120 participants with complete data for all five principal outcomes. Sensitivity analyses retained all 121 source records to assess the consequences of this exclusion.

### 2.2. Measures

**Video Gaming Activity** 

Video gaming activity was assessed using a brief self-report form. Participants reported whether they currently played video games and indicated their typical gaming intensity, gaming history, gaming tenure, preferred gaming devices, and game genres. Current gaming status was coded as a binary variable distinguishing adolescents who currently played from those who did not currently play and served as the principal predictor. The non-current group included both former players and adolescents who had never played; accordingly, this group is described as non-current players rather than as non-gamers. Current gaming status is interpreted as a measure of current behavioral status rather than as a proxy for the presence or absence of lifetime gaming exposure.

Current status followed the participant’s questionnaire response about currently playing video games; former players reported previous play without current participation, and never-players reported no previous play. The questionnaire did not specify a fixed reference period for current play or a minimum inactivity interval for former play. These categories therefore represent self-classified status rather than a standardized recency threshold.

For exploratory analyses, gaming intensity was represented by three categories: non-current play, current play up to and including 1 h/day, and current play for more than 1 h/day. During administration, the investigator clarified that the questionnaire’s “1–4 h” category referred to more than 1 h and up to 4 h/day. The higher-intensity category combined 37 participants in this category with five reporting more than 4 h/day. Among current players, a separate comparison contrasted >1 h/day with ≤1 h/day. Broad self-report categories and the small highest-intensity subgroup limited assessment of a dose–response association. Gaming tenure was recorded in categories labelled less than 1 year, 1–5 years, and 5–10 years. The tenure analysis combined the first two categories and compared them with the recorded 5–10-year category; exact years were unavailable, so this is not a precisely defined five-year cutoff.

**Cognitive and Psychomotor Tasks** 

Cognitive and psychomotor performance was assessed using selected computerized tasks from the Integrated System for the Measurement of Psychological Variables (SDP) produced by GPE Psychotronics (Poland). The reliability and convergent validity of the SDP measures, including the Number Localization Test and the simple and complex coordination tasks, have been examined in a large psychometric study [22]. The system comprises computer-based tasks designed to assess visual search, perceptual accuracy, visuomotor coordination, response precision, and information-processing speed and has been described in previous methodological and empirical work [12,22,23,24]. The present study included the Line Test, Number Localization Test, Simple Coordination Test, and Complex Coordination Test.

**Simple Coordination Test** 

The Simple Coordination Test assessed basic visuomotor coordination and speeded visuomotor responding. Successive digits were displayed on the computer screen, and participants responded by pressing the corresponding key. The task lasted 60 s and was performed in forced mode, in which the next stimulus was presented only after a correct response. Mean response time and the number of correct responses were recorded. Shorter response times and more correct responses indicated more efficient performance. Because the task was administered in forced mode, the correct-response count represents throughput (the number of correct trials completed) rather than an independent measure of accuracy.

**Complex Coordination Test** 

The Complex Coordination Test assessed visuomotor coordination under greater cognitive demands. Participants were presented with simple arithmetic operations involving addition or subtraction of single-digit numbers and entered the correct result using the keyboard. The task lasted 60 s. Mean response time and the number of correct responses were recorded. Shorter response times and more correct responses indicated more efficient performance. As in the Simple Coordination Test, the correct-response count is interpreted as a throughput measure rather than conventional response accuracy because task progression depended on a correct response.

**Psychomotor Throughput (PiM)** 

Psychomotor throughput was represented by the PiM index, defined as the sum of correct responses obtained in the Simple and Complex Coordination Tests. Higher PiM values indicated greater throughput in tasks requiring rapid visuomotor coordination, response precision, and elementary cognitive processing. Previous research has demonstrated a positive association between PiM and Raven’s matrix performance, supporting its relevance as an indicator of cognitive–motor efficiency [11]. PiM was recalculated from its two constituent scores for every participant to ensure uniform scoring. In the present study, PiM is therefore interpreted as a composite cognitive–motor throughput index, not as an independent measure of accuracy or as a broad index of generalized cognitive ability.

**Number Localization Test** 

The Number Localization Test was used as an indicator of visual-search efficiency and perceptual processing speed. Participants memorized target numbers and subsequently located them among competing numerical stimuli presented on the screen. Completion time was the principal performance indicator, with shorter times reflecting more efficient visual scanning and information processing.

**Line Test** 

The Line Test assessed perceptual accuracy and visual attention. Participants viewed successive displays containing thin lines, determined their number, and entered the corresponding response. The task comprised nine displays. Completion time and number of correct responses were analyzed separately. Considering both indicators allowed faster performance to be interpreted in the context of response accuracy rather than assuming that speed alone represented superior performance.

**Children’s Color Trails Test** 

Sequential–executive processing was assessed using the Children’s Color Trails Test—distributed by Pracownia Testów Psychologicznych PTP (Poland), a neuropsychological test designed for individuals aged 8–17 years [25]. The primary analysis focused on CCTT-2, in which participants connected numbered stimuli in ascending order while alternating between two colors, thereby imposing demands on attentional switching, sequencing, self-monitoring, and visual–motor performance. Completion time was the primary indicator; shorter times indicated more efficient performance. CCTT-1 completion time and the logarithmic CCTT-2/CCTT-1 time ratio were examined in supplementary analyses. CCTT completion times were recorded in minutes.

**School Achievement** 

School achievement was assessed using self-reported average grades. Participants selected the interval corresponding to their overall grade average, and the midpoint of the selected interval was used as a quantitative indicator. Because grades were self-reported and recorded in predefined intervals, school achievement was treated as an exploratory outcome.

### 2.3. Procedure and Ethical Considerations

The preliminary study methodology and organizational procedures were developed between September 2022 and February 2023 for implementation in six public secondary schools in southern Poland. During this preparatory phase, school authorities were informed about the study objectives, and organizational arrangements for the planned research were established. The research project was subsequently submitted for formal ethical review. A favorable ethical opinion was issued on 14 June 2023 by the Research Ethics Committee of the University of Business—National Louis University in Nowy Sącz, Poland (Resolution No. 1/2022/2023). All participant assessments and research data collection were conducted after the favorable ethical opinion had been obtained, in accordance with the approved research procedures and institutional permissions. Written informed consent was obtained from parents or legal guardians, and participating adolescents additionally provided assent. Participation was voluntary and anonymous.

Assessments were conducted individually during regular school hours, always in the morning, in a quiet, unoccupied classroom under favorable ergonomic conditions. Participants first provided demographic, school-related, and video gaming information and subsequently completed the Children’s Color Trails Test (CCTT), followed by the computerized tasks from the Integrated System for the Measurement of Psychological Variables (SDP). Standardized task instructions were displayed on the computer screen, with verbal clarification provided by the examiner when required. Each assessment session lasted approximately 15–20 min per participant. A school counsellor or teacher was present throughout the assessment procedure. Participants were informed that they could discontinue their participation at any time without consequences. The recruitment and consent procedures of the original school-based study have also been described in a previous publication examining related cognitive and psychomotor outcomes [12].

The study was non-invasive, did not involve clinical intervention, and did not include the collection of sensitive medical data. Data were used exclusively for scientific purposes and analyzed at the group level. The present study represents a secondary, cross-sectional reanalysis of an existing dataset collected within the scope of the original school-based research project, rather than a separate prospective investigation. No additional participants were recruited, and no new data were collected for the present analysis.

### 2.4. Data Preparation

The dataset was examined for completeness, coding consistency, and implausible values before inferential analysis. The final database contained no missing observations for the five principal outcomes. PiM was recalculated as the sum of correct responses in the Simple and Complex Coordination Tests. This procedure identified two stored PiM values that were inconsistent with the defined scoring rule; the recalculated values were used in all analyses.

The two discrepant stored PiM scores were 108 instead of 106 and 90 instead of 100. Analytical files retained the source measurements and used the uniformly recalculated PiM score.

Mean response time and correct-response count were strongly inversely related within the Simple Coordination Test, r = −0.989, and Complex Coordination Test, r = −0.961. This pattern reflected the forced-mode administration, in which faster correct responding allowed more items to be completed. These variables were therefore treated as complementary component-level indicators rather than independent primary endpoints. Given this task structure, correct-response counts were interpreted as throughput indicators and not as independent accuracy endpoints.

PiM directly includes both correct-response counts. Component analyses consequently describe the structure of the composite and overlapping task performance; they are not independent converging evidence for PiM.

Time-based outcomes were positively bounded and right-skewed to varying degrees. Number Localization, Line Test, CCTT-1, CCTT-2, and coordination response times were therefore logarithmically transformed for regression analyses; raw values were retained for descriptive presentation. PiM, coordination correct-response counts, and Line Test accuracy were analyzed on their original scales.

### 2.5. Statistical Analysis

Descriptive statistics were calculated for demographic characteristics, gaming variables, and all performance outcomes. The association between sex and current gaming status was evaluated using Pearson’s chi-square test, with Cramer’s V as the effect-size measure. Unadjusted differences between current and non-current players were quantified using Hedges’ g.

Separate multiple linear regression models were estimated for five principal outcomes: PiM, Number Localization completion time, Line Test completion time, Line Test accuracy, and CCTT-2 completion time. Current gaming status was the predictor of interest, and age and sex were included as covariates. Heteroskedasticity-consistent HC3 standard errors were used [26]. Pointwise 95% confidence intervals and two-sided tests used the residual t distribution. For logarithmically transformed outcomes, 100 × (exp(B) − 1) expresses the adjusted percentage difference in geometric mean time; the confidence limits were transformed in the same way. With only two non-current boys, the full-sample coefficient depends on an additive model and cannot provide a reliable within-boy association.

The Benjamini–Hochberg procedure [27] controlled the false discovery rate across the five principal outcomes. A separate Benjamini–Hochberg correction was applied across four supplementary coordination outcomes: mean response time and correct-response count in the Simple and Complex Coordination Tests. Because only two boys were non-current players, the five principal models were repeated in girls only, with age as the covariate; false discovery rate correction was again applied across five outcomes. Because the sample contained only two non-current boys, the girls-only models were treated as an essential sensitivity analysis for evaluating whether the principal pattern was present within a sex subgroup that contained adequate numbers in both gaming-status categories.

The female-only analysis is a sensitivity analysis within the same dataset, not an independent replication. Boys-only models and a gaming-by-sex interaction were not estimated.

Exploratory intensity models used ordinary least squares with HC3 covariance, adjusting for age and sex. Omnibus tests were corrected across five outcomes, and 15 pairwise contrasts formed a separate family. The within-player comparison of >1 with ≤1 h/day was corrected across five outcomes. Gaming history was examined among girls with age adjustment, correcting five omnibus tests and 15 pairwise contrasts separately. Tenure models included 91 current players with available tenure data and adjusted for age, sex, and intensity, with correction across five outcomes. Seven multiple-response genre indicators were each modelled with age, sex, and intensity across the five outcomes; correction covered 35 tests. School achievement was analysed separately. All tests were two-sided.

School membership was reconstructed retrospectively by the investigator from school visits and questionnaire dates and linked to checked source records using codes S1–S6. Sensitivity models added school fixed effects to the age- and sex-adjusted specification; female-only models adjusted for age and school. We also used conventional school-clustered CR1 covariance with multiplier G/(G − 1) × (N − 1)/(N − K), where G is the number of schools and K the number of regression coefficients, and t inference with G − 1 degrees of freedom [28]. Six schools were represented overall and five among girls. With so few, unequal clusters, these clustered tests may be unreliable and are exploratory; fixed effects alone do not account for within-school dependence.

Additional sensitivity analyses retained all 121 source records and omitted one school at a time. Benjamini–Hochberg correction covered five outcomes separately within each sample and model specification, rather than all sensitivity specifications jointly. These analyses assess dependence on modelling and sample choices and do not constitute independent confirmation.

All analyses were conducted using reproducible scripts written in Python 3.13.5, with NumPy, pandas, and SciPy libraries. Rerunning the archived scripts reproduced 17 CSV output files. The five principal age- and sex-adjusted HC3 models were also reproduced in R 4.5.0 using the Rj Editor 2.7.18 within jamovi. Coefficients, HC3 standard errors, confidence intervals, *p* values, and Benjamini–Hochberg-adjusted values agreed with Python to the reported precision. The separate R verification concerned these five principal models, not the supplementary analyses.

No a priori power calculation was conducted for the present research questions because the sample was determined by the available dataset. Inferential emphasis was placed on the five principal outcomes, confidence intervals, effect sizes, and multiplicity-adjusted *q* values. All analyses remain exploratory; correction within reporting families does not make this secondary reanalysis confirmatory.

## 3. Results

### 3.1. Sample Characteristics and Current Gaming Status

Of the 120 adolescents, 92 (76.7%) reported currently playing video games and 28 (23.3%) did not currently play. Current gaming status differed markedly by sex, χ^2^(1) = 26.83, *p* < 0.001, Cramer’s *V* = 0.473. Fifty-eight of 60 boys (96.7%) were current players, compared with 34 of 60 girls (56.7%). The non-current group comprised 20 former players and eight adolescents who had never played. Age and sex were retained as covariates in all principal full-sample models.

### 3.2. Principal Cognitive and Psychomotor Outcomes

Descriptive statistics, unadjusted effect sizes, and adjusted associations for the five principal outcomes are presented in Table 2.

### 3.3. Psychomotor Throughput (PiM)

After adjustment for age and sex, current gaming was associated with a 6.92-point higher PiM score, 95% CI [1.24, 12.59], *p* = 0.017, *q* = 0.043. The complete model explained 7.3% of the variance, and current gaming added 3.9 percentage points beyond age and sex.

### 3.4. Visual-Search Efficiency

In the adjusted model, current gaming was associated with a 9.6% shorter geometric mean Number Localization completion time, 95% CI [2.6%, 16.1% shorter], *p* = 0.008, *q* = 0.042. The model explained 8.2% of the variance, with current gaming contributing an additional 4.6 percentage points beyond age and sex.

### 3.5. Perceptual Speed and Accuracy

The adjusted Line Test time difference corresponded to a 10.4% shorter geometric mean completion time, 95% CI [0.7%, 19.1% shorter], *p* = 0.036. This association did not meet the false-discovery-rate threshold, *q* = 0.060. Current gaming added 3.2 percentage points to explained variance beyond age and sex.

Line Test accuracy did not differ significantly between current and non-current players, adjusted *B* = 0.28, 95% CI [−0.69, 1.25], *p* = 0.567, *q* = 0.567. Thus, faster performance was not accompanied by a detected reduction in accuracy; however, the confidence interval did not establish statistical equivalence between groups.

### 3.6. Sequential–Executive Processing

For CCTT-2, current gaming was associated with a 9.8% shorter geometric mean time, but the confidence interval included the null, 95% CI [22.0% shorter, 4.3% longer], *p* = 0.163, *q* = 0.204. CCTT-1 time was also inconclusive, 9.4% shorter, 95% CI [23.6% shorter, 7.4% longer], *p* = 0.251. The log CCTT-2/CCTT-1 time ratio provided insufficient evidence of a group difference, *p* = 0.959.

Each additional year of age was associated with an estimated 10.4% shorter geometric mean CCTT-2 completion time, 95% CI [4.1%, 16.4% shorter], *p* = 0.002. No direct comparison of the age and gaming associations was performed.

### 3.7. Component-Level Coordination Performance

Supplementary models examined the two components underlying PiM (Table 3).

In the Simple Coordination Test, current players had 6.1% shorter mean response times, *p* = 0.006, *q* = 0.015, and completed 4.29 more correct trials, *p* = 0.007, *q* = 0.015. Both effects were moderate in unadjusted magnitude (|*g*| = 0.57). In the Complex Coordination Test, differences favored current players descriptively but were not statistically significant after adjustment: mean response time, *p* = 0.193, and correct responses, *p* = 0.137. The PiM association was most clearly reflected in simple-coordination performance. These component results overlap with PiM and do not independently confirm it. Significance for one component and nonsignificance for another do not establish a difference between associations; no direct coefficient contrast was tested.

### 3.8. Sensitivity Analysis in Girls

Because only two boys were non-current players, the five principal analyses were repeated among girls (n = 60; 34 current and 26 non-current players). This analysis presented in Table 4 therefore provides the clearest within-sex estimate of the association available in the present dataset.

The female-only analysis showed a similar principal pattern within the same dataset. Current gaming was associated with higher PiM, B = 7.12, 95% CI [0.98, 13.26], *p* = 0.024, *q* = 0.040; a 9.9% shorter Number Localization time, *p* = 0.015, *q* = 0.040; and a 12.8% shorter Line Test time, *p* = 0.016, *q* = 0.040. Line Test accuracy and CCTT-2 time remained inconclusive. These findings reduce concern about a purely between-sex explanation, but they are not an independent replication and do not provide a reliable estimate for boys.

School-adjusted inference differed for the time outcomes, as described in Section 3.11 and Table S2.

### 3.9. Exploratory Analyses of Gaming Intensity

Three-level intensity models compared non-current players (n = 28), current players reporting up to 1 h/day (n = 50), and current players reporting more than 1 h/day (n = 42). Table 5 also presents the direct dose comparison between regular and sporadic current players.

The three-level omnibus tests were nominally significant for Number Localization time, *p* = 0.031, and borderline for PiM, *p* = 0.060, and Line Test time, *p* = 0.050. None remained significant after false-discovery-rate correction; the smallest adjusted value was *q* = 0.099. Exploratory contrasts suggested that both sporadic and regular players tended to outperform non-current players, whereas regular players did not outperform sporadic players. None of the 15 pairwise contrasts survived correction; the smallest *q* was 0.106.

The direct comparison within current players provided insufficient evidence of a dose–response relation. PiM means were nearly identical among regular and sporadic players, M = 102.05 and 102.02, respectively. No within-player contrast approached statistical significance after correction (all *q* ≥ 0.583). Given the broad self-report categories and modest subgroup sizes, this null result should be interpreted as an absence of detected dose–response evidence in the present dataset rather than as evidence that gaming intensity is unrelated to performance.

### 3.10. Additional Exploratory Analyses

**Gaming History** 

Gaming history was examined among girls because all eight participants who had never played were girls. The female subsample comprised 34 current players, 18 former players, and eight never-players. No omnibus association survived correction across five outcomes (smallest *q* = 0.126). Current players were nominally faster than former players in Number Localization (9.9% shorter, *p* = 0.043) and the Line Test (13.9% shorter, *p* = 0.028), but neither contrast survived correction across the 15 pairwise tests (both *q* = 0.212). The small never-player group precluded firm conclusions about lifetime gaming history.

**Gaming Tenure and Genre** 

Among current players, the recorded 5–10-year tenure category was compared with the combined less-than-1-year and 1–5-year categories (n = 91 with available tenure data), adjusting for age, sex, and gaming intensity. No principal outcome was associated with tenure (all *q* ≥ 0.844). Seven multiple-response genre indicators were then examined across the five principal outcomes, with adjustment for age, sex, and intensity. None of the 35 genre associations survived correction (smallest *q* = 0.261). Thus, the data did not support conclusions about differential cognitive performance by game genre.

**School Achievement** 

Mean self-reported school grade was 3.76 (*SD* = 0.61) among current players and 3.98 (*SD* = 0.58) among non-current players. After adjustment for age and sex, current gaming was not significantly associated with school achievement, *B* = −0.23, 95% CI [−0.54, 0.08], *p* = 0.148. The association was weaker in the female-only analysis, *B* = −0.14, 95% CI [−0.48, 0.19], *p* = 0.397.

### 3.11. School and Sample Sensitivity Analyses

The distribution across six schools was unequal (Table S1). School fixed-effects models with HC3 standard errors retained the positive PiM association in the principal full sample (B = 8.05, 95% CI [2.53, 13.57], *q* = 0.023) and among girls (B = 9.21, 95% CI [3.39, 15.04], *q* = 0.013). Number Localization met the correction threshold in the full sample (9.5% shorter, *q* = 0.033), but not among girls (*q* = 0.062); female Line Test time also did not meet the threshold (*q* = 0.085; Table S2).

Analyses retaining all 121 source records and exploratory school-clustered analyses are reported in Tables S3 and S4. Some inferences varied with sample and covariance specification. Omitting individual schools retained positive PiM estimates of 7.11–8.89 points overall and 8.16–10.25 among girls, but *q* ranged from 0.016 to 0.070 and from 0.008 to 0.177, respectively. Thus, not every omission model met the correction threshold. Number Localization estimates remained negative but varied in precision, and CCTT-2 estimates were particularly sensitive to school omission (Table S5). Directional consistency should therefore be distinguished from consistently meeting a significance threshold.

## 4. Discussion

The present study examined whether current video gaming among adolescents aged 14–16 years was associated with differences in visual–perceptual and psychomotor performance. After adjustment for age and sex and application of the Benjamini–Hochberg procedure, current players obtained higher PiM scores and shorter Number Localization times in the principal models. PiM estimates remained positive across school and sample sensitivity analyses, although not every leave-one-school-out model met the correction threshold. Inference for visual-search time was more sensitive to specification. Line Test time did not meet the principal FDR threshold, and Line Test accuracy and CCTT-2 estimates were inconclusive. Simple coordination most clearly reflected the PiM association, but the overlapping component analyses do not independently confirm it. The female-only comparison reduces concern about a purely between-sex explanation, while providing no reliable estimate for boys.

The first major finding was higher psychomotor throughput among current players. The association remained significant after adjustment for age and sex and correction for multiple comparisons. This finding was consistent with previous research indicating more favorable indices of reaction time, visual processing, and visuomotor coordination among video game players [4,5,10,11]. A particularly relevant point of comparison was provided by Szabo et al., who found that regular League of Legends players exhibited shorter reaction times, faster target acquisition, and more favorable selected indices of visuomotor coordination. The convergence between these findings therefore concerned performance requiring rapid integration of visual input with motor responding, rather than broadly defined cognitive ability. Because current gaming status was strongly associated with sex, the full-sample coefficient should be interpreted together with the girls-only sensitivity analysis rather than as a sex-independent estimate applicable equally to boys and girls [10].

The two components underlying PiM described the structure of this association. In the Simple Coordination Test, current players responded approximately 6.1% faster and completed 4.29 more correct trials than non-current players; both effects remained significant after FDR correction. In the Complex Coordination Test, the direction of group differences was similar, although the effects did not reach statistical significance after covariate adjustment. This pattern is compatible with a role for rapid stimulus detection and direct motor responding, but no direct contrast tested whether associations differed between tasks. A similar domain-specific pattern has been reported in studies of reaction time, visual attention, and visuomotor coordination, in which player advantages did not extend uniformly across all measured components of performance [3,5,10,29,30]. The present findings therefore do not establish uniformly superior psychomotor functioning or a stronger association with one component than another. In forced mode, this count reflects the amount of correctly completed material within the task rather than an independent accuracy dimension, supporting interpretation of PiM primarily as cognitive–motor throughput.

The counts contribute directly to PiM, and their near-inverse relation with mean response times follows the fixed-duration task structure. They should not be treated as independent convergent evidence.

A second major finding concerned visual-search efficiency. Current players completed the Number Localization Test approximately 9.6% faster after adjustment for age and sex, and the effect remained significant after FDR correction. A particularly relevant point of reference was the study by Argilés et al., who examined adolescents of nearly identical age. Regular action video game players completed the horizontal condition of the Developmental Eye Movement Test and the King–Devick Test more quickly, both of which involve sequential visual search and rapid processing of visual information [5]. The importance of selective visual attention, rapid target localization, and efficient information processing has also been reported in other contemporary studies and meta-analyses of action video gaming [3,10,29,30]. The convergence with the Number Localization Test was noteworthy because a similar pattern was observed across independent samples and different assessment methods. Current players also completed the Line Test approximately 10.4% faster after covariate adjustment; however, this association did not remain significant after FDR correction (*q* = 0.060). It therefore did not provide additional statistically conclusive evidence of greater perceptual speed, although its direction was consistent with both the Number Localization Test findings and the girls-only analysis. School-adjusted and clustered analyses qualify the stability of the visual-search finding. Differences in significance across tasks do not themselves demonstrate domain specificity, although the literature provides context for investigating that possibility [3,5,29]. Although the association with Number Localization time reached the false-discovery-rate threshold in the principal model, its statistical support was less consistent across sensitivity analyses than that observed for psychomotor throughput. Accordingly, the visual-search finding should be regarded as exploratory and more sensitive to analytical specification, rather than as equally robust evidence of a gaming-related performance difference.

An important component of interpretation involved distinguishing processing speed from response accuracy. No significant group difference was observed in the number of correct responses on the Line Test. Thus, the shorter completion time among current players was not accompanied by a detected reduction in accuracy, although the width of the confidence interval did not permit a conclusion of statistical equivalence between groups. This pattern differed in part from the findings of Özçetin et al., who reported faster but less accurate performance among adolescents with more intensive gaming experience [16]. In contrast, Szabo et al. observed shorter reaction times among players on the Simon Task without a significant between-group difference in accuracy [10]. More recent work on selective attention has likewise suggested that associations between gaming experience, response speed, and accuracy may vary as a function of task characteristics and perceptual load [3]. The present findings neither establish equivalence in accuracy nor rule out a speed–accuracy trade-off.

A different pattern emerged for more complex sequential–executive processing. Current gaming status was not significantly associated with completion time on the Children’s Color Trails Test (CCTT-2), nor with CCTT-1 or the CCTT-2/CCTT-1 ratio. Age was associated with CCTT-2 performance: each additional year of age was associated with an estimated 10.4% shorter completion time. These models did not directly test whether age and gaming associations differed in strength. A possible interpretation concerns processes proximal to dynamic gameplay, including rapid visual selection, response execution, and coordination; however, inconclusive CCTT estimates cannot establish the absence of an executive association. This interpretation was consistent with evidence suggesting domain-specific rather than global associations between gaming and cognitive functioning [4,14,15,30]. Related context has also been provided by recent neuroimaging data. Nagata et al., analyzing 5686 adolescents from the Adolescent Brain Cognitive Development Study, reported heterogeneous associations between gaming behaviors and cortical thickness and surface area depending on the gaming characteristic examined [6]. Although these findings did not directly explain the present behavioral results, they were compatible with the broader interpretation of selective, domain-specific correlates of gaming rather than a single generalized cognitive advantage [7,8,31].

The exploratory analyses likewise provided insufficient evidence for an association between greater self-reported gaming intensity and better test performance. After correction for multiple comparisons, no significant effect of gaming intensity was observed, and among current players, those reporting more than one hour of gaming per day did not outperform those reporting up to one hour per day. No significant associations were found for gaming tenure or the analyzed game-genre categories. These results do not establish that current status is more informative than intensity or that a dose–response association is absent. The earlier report from the same dataset also considered gaming intensity; it is not independent evidence. More broadly, more recent research has suggested that gaming duration alone may be an insufficient indicator of cognitive correlates, which may additionally depend on the characteristics of the activity, game type, and dysregulated patterns of gaming behavior [4,12,29]. The absence of genre effects in the present analyses should nevertheless be interpreted as insufficient evidence for differences rather than as evidence that genre is irrelevant, particularly because several genre categories were small, participants could endorse multiple categories, and the analyses required correction across a large number of tests. These null intensity findings are necessarily tentative because exposure was measured in broad self-report categories and the most intensive gaming category was very small.

In contrast to the earlier unadjusted report [12], none of the 35 adjusted genre associations met the present correction threshold. Covariate adjustment, testing families, and other analytical choices changed together, so this difference cannot be attributed to one decision in isolation.

A major strength of the present study was the simultaneous assessment of several levels of functioning, including psychomotor throughput, simple and more complex components of coordination, visual-search speed, perceptual speed and accuracy, and sequential–executive processing. This allowed for a description of associations across tasks, although no formal contrast of domain-specific associations was performed. Speed and accuracy were also analyzed separately, age and sex were controlled, and false-discovery-rate correction was applied. The regression models additionally used heteroskedasticity-consistent HC3 standard errors, meaning that estimation of coefficient uncertainty did not depend on the strict assumption of equal residual variance across observations. The sensitivity analysis restricted to girls showed a similar principal pattern within the same sample, which was particularly important given the strong association between sex and current gaming status.

The study also had several important limitations. First, the cross-sectional design precluded causal inference. As in the studies by Argilés et al. and Szabo et al., it was not possible to determine whether gaming experience contributed to greater visual–psychomotor efficiency or whether adolescents with more favorable baseline levels of processing speed and coordination were more likely to engage in gaming. Both mechanisms may also have operated simultaneously [5,10]. Similar limitations have been emphasized by Montolio-Vila et al. [13].

Familiarity with computers and keyboard responses could also contribute to SDP performance. Preferred devices were recorded, but proficiency with the response interface was not separately measured. The present analyses cannot distinguish transferable ability from interface familiarity or other unmeasured differences.

Second, gaming activity was assessed using self-report. The primary predictor was intentionally based on a simple distinction between adolescents who currently played and those who did not currently play, which did not capture the full heterogeneity of gaming experience. Moreover, the non-current group included 20 former players and only eight participants who had never played. This limited the extent to which the comparison could be interpreted as a conventional contrast between “gamers” and individuals with no previous gaming experience and complicated interpretation of between-group differences. Accordingly, the primary comparison should be understood as current versus non-current gaming status, not as gaming exposure versus no lifetime exposure. The broad self-report intensity categories also limited the precision of any dose–response analysis.

The absence of a fixed current-gaming reference window or a recorded cessation interval further limits recency-based interpretations.

Third, current gaming status was strongly associated with sex, as almost all boys belonged to the current-player group. Although sex was included as a covariate and the principal analyses were additionally repeated among girls only, the sample composition did not permit a reliable separate estimate of the gaming association among boys. In addition, several categories related to gaming intensity, gaming experience, and game genre included relatively small numbers of participants, which limited statistical power to detect more subtle associations. The influence of unmeasured confounding variables, such as participation in other activities requiring high levels of visuomotor skill, could also not be excluded. This imbalance means that statistical adjustment for sex cannot create information that is absent from the data: the study contains almost no non-current male comparison group. The girls-only analysis therefore provides the strongest within-sex sensitivity evidence, whereas the magnitude of the association among boys remains undetermined.

Finally, several secondary and exploratory subgroup analyses were based on small cell sizes. Nonsignificant findings for complex coordination, CCTT performance, gaming tenure, intensity, or genre should therefore not be interpreted as evidence of equivalence or proof of no association.

School membership was reconstructed retrospectively, and the small number of unequal schools limits conventional clustered inference [28]. Fixed effects address school mean differences but do not remove within-school dependence. School-omission analyses show that some inferences depend on individual schools. Availability-based recruitment and missing invitation and refusal counts also limit assessment of representativeness. Finally, the unregistered secondary analysis of previously reported, correlated outcomes remains exploratory despite correction within specified families.

## 5. Conclusions

Theoretical Conclusions. The findings indicate that current video gaming was selectively associated with certain aspects of visual–psychomotor functioning rather than with a generalized cognitive advantage. The most consistent association was observed for psychomotor throughput (PiM), reflecting the number of correctly completed responses in timed coordination tasks. Component-level analyses characterized the performance structure underlying PiM and should not be interpreted as independent confirmation of this association. Although visual-search performance generally favored current players, the statistical evidence for this association was less consistent across sensitivity analyses and analytical specifications. The findings therefore support a cautious, domain-specific interpretation of the observed associations, consistent with previous research emphasizing the heterogeneous cognitive and psychomotor correlates of video gaming [4,5,6,10]. Given the exploratory, cross-sectional nature of the study, these results do not establish causality, a generalized cognitive advantage, or a definitive hierarchy of associations across cognitive domains.

Cognitive Conclusions. From a cognitive perspective, the clearest association concerned performance efficiency in tasks requiring rapid visuomotor responding, as reflected by higher PiM scores among current players. The Number Localization Test also indicated faster visual-search performance in the principal age- and sex-adjusted model; however, this finding was less stable across sensitivity analyses and should therefore be interpreted as preliminary. No statistically conclusive associations were identified for Line Test accuracy or sequential–executive processing assessed using the CCTT-2. Furthermore, the component-level coordination measures overlapped with the PiM composite and did not provide independent evidence of additional cognitive advantages. Taken together, these findings suggest that the observed differences were concentrated primarily in psychomotor throughput, with less consistent evidence for visual-search efficiency, rather than reflecting broadly superior cognitive functioning among current video game players.

Practical Conclusions. From a practical perspective, the findings may inform future investigations into the relationship between digital activities and specific aspects of visual–psychomotor performance, particularly tasks requiring rapid stimulus processing and coordinated motor responses. However, the results do not justify recommending video gaming as a method of cognitive training or assuming that greater gaming exposure produces better cognitive performance. The cross-sectional design precludes conclusions about the direction or causality of the observed associations, while the exploratory analyses did not identify clear dose–response relationships involving gaming intensity, tenure, or game genre. Future research should prioritize longitudinal and experimental designs, objective and standardized measures of gaming exposure, and more precise characterization of the cognitive demands associated with different types of games. Larger and more balanced samples, together with appropriate consideration of school-level clustering and potential confounding factors, will be essential for determining whether the observed associations are reproducible and whether they reflect gaming-related experience, pre-existing individual differences, or both.

## Figures and Tables

**Table 1:** Sample Characteristics and Video Gaming Activity.

Variable	n	%
Sex		
Girls	60	50.0
Boys	60	50.0
Age		
14 years	27	22.5
15 years	67	55.8
16 years	26	21.7
Current video gaming status		
Does not currently play	28	23.3
Currently plays	92	76.7
Gaming history among non-current players		
Never played	8	28.6
Former player	20	71.4
Gaming intensity among current players		
Up to 1 h/day	50	54.3
More than 1 to 4 h/day	37	40.2
More than 4 h/day	5	5.4
Gaming tenure among current players		
Less than 1 year	4	4.3
1–5 years	34	37.0
5–10 years	53	57.6
Missing	1	1.1

Percentages for gaming history were calculated within non-current players (n = 28). Percentages for gaming intensity and tenure were calculated within current players (n = 92).

**Table 2:** Current Video Gaming Status and Principal Cognitive and Psychomotor Outcomes.

Outcome	Current M (SD)	Non-Current M (SD)	*g*	Adjusted Effect [95% CI]	*p*/*q*
PiM	102.03 (13.14)	94.04 (10.96)	0.63	B = 6.92 [1.24, 12.59]	*p* = 0.017 *q* = 0.043
Number Localization time	6.07 (1.06)	6.80 (1.10)	−0.67	−9.6% [−16.1%, −2.6%]	*p* = 0.008 *q* = 0.042
Line Test time	7.83 (1.83)	8.98 (1.82)	−0.62	−10.4% [−19.1%, −0.7%]	*p* = 0.036 *q* = 0.060
Line Test accuracy	6.65 (1.95)	6.36 (2.08)	0.15	B = 0.28 [−0.69, 1.25]	*p* = 0.567 *q* = 0.567
CCTT-2 time	0.360 (0.151)	0.389 (0.093)	−0.21	−9.8% [−22.0%, 4.3%]	*p* = 0.163 *q* = 0.204

N = 120. Hedges’ g is unadjusted. Adjusted effects are regression estimates from models controlling for age and sex and using HC3 robust standard errors with a t reference distribution. Time outcomes were logarithmically transformed; negative percentages indicate shorter geometric mean times among current players. Number Localization and Line Test times are reported in seconds; CCTT-2 time is reported in minutes. *q* values are Benjamini–Hochberg-adjusted across the five principal outcomes. Confidence intervals are pointwise.

**Table 3:** Current Video Gaming Status and Component-Level Coordination Performance.

Outcome	Current M (SD)	Non-Current M (SD)	*g*	Adjusted Effect [95% CI]	*p*/*q*
Simple Coordination mean RT	0.879 (0.090)	0.930 (0.082)	−0.57	−6.1% [−10.1%, −1.8%]	*p* = 0.006 *q* = 0.015
Simple Coordination correct trials	66.66 (7.05)	62.71 (6.23)	0.57	B = 4.29 [1.17, 7.41]	*p* = 0.007 *q* = 0.015
Complex Coordination mean RT	1.718 (0.417)	1.934 (0.479)	−0.50	−7.1% [−16.8%, 3.8%]	*p* = 0.193 *q* = 0.193
Complex Coordination correct trials	35.37 (7.85)	31.32 (6.98)	0.53	B = 2.63 [−0.84, 6.09]	*p* = 0.137 *q* = 0.182

Hedges’ g is unadjusted. Adjusted effects are from models controlling for age and sex and using HC3 robust standard errors. Mean response times (RTs) are reported in seconds and were logarithmically transformed. Correct-trial counts represent throughput under forced-mode administration rather than an independent accuracy measure. *q* values are Benjamini–Hochberg-adjusted across the four component outcomes. Confidence intervals are pointwise.

**Table 4:** Sensitivity Analysis of Principal Outcomes in Girls.

Outcome	Current M (SD)	Non-Current M (SD)	Adjusted Effect [95% CI]	*p*/*q*
PiM	100.56 (13.16)	94.12 (11.29)	B = 7.12 [0.98, 13.26]	*p* = 0.024 *q* = 0.040
Number Localization time	6.16 (0.96)	6.81 (1.14)	−9.9% [−17.1%, −2.1%]	*p* = 0.015 *q* = 0.040
Line Test time	8.00 (1.93)	9.14 (1.78)	−12.8% [−21.9%, −2.6%]	*p* = 0.016 *q* = 0.040
Line Test accuracy	6.65 (1.81)	6.31 (2.13)	B = 0.31 [−0.77, 1.39]	*p* = 0.568 *q* = 0.568
CCTT-2 time	0.379 (0.195)	0.389 (0.095)	−10.5% [−24.0%, 5.5%]	*p* = 0.183 *q* = 0.229

n = 60. Models were adjusted for age and used HC3 robust standard errors. Time outcomes were logarithmically transformed. Number Localization and Line Test times are reported in seconds; CCTT-2 time is reported in minutes. *q* values are Benjamini–Hochberg-adjusted across the five outcomes. Confidence intervals are pointwise.

**Table 5:** Exploratory Associations with Gaming Intensity.

Outcome	F(2, 115)	Omnibus *p*/*q*	Regular vs. Sporadic Effect [95% CI]	Contrast *p*/*q*
PiM	2.89	*p* = 0.060 *q* = 0.099	B = −1.30 [−7.24, 4.63]	*p* = 0.664 *q* = 0.830
Number Localization time	3.57	*p* = 0.031 *q* = 0.099	−0.2% [−9.1%, 9.6%]	*p* = 0.967 *q* = 0.967
Line Test time	3.07	*p* = 0.050 *q* = 0.099	+7.4% [−3.7%, 19.7%]	*p* = 0.198 *q* = 0.583
Line Test accuracy	0.77	*p* = 0.463 *q* = 0.463	B = 0.51 [−0.33, 1.34]	*p* = 0.233 *q* = 0.583
CCTT-2 time	1.26	*p* = 0.289 *q* = 0.361	−2.9% [−14.1%, 9.7%]	*p* = 0.629 *q* = 0.830

Three-level models were adjusted for age and sex. The within-current-player contrast compared more than 1 h/day with up to 1 h/day. Positive time percentages indicate longer geometric mean times in regular players. Omnibus *q* values were adjusted across five tests; within-player *q* values were adjusted across five contrasts. Confidence intervals are pointwise.

## Data Availability

The individual-level data supporting the findings of this study are not publicly available due to privacy and ethical considerations associated with research involving minors. De-identified analytical data may be made available by the corresponding author upon reasonable request, subject to applicable ethical and data protection requirements. Information regarding the study questionnaire and assessment procedures is available from the corresponding author upon reasonable request. Access to standardized psychological tests and other copyrighted materials is subject to the licensing conditions and restrictions established by the respective publishers or rights holders.

## Supplementary Materials

The following supporting information can be downloaded at: https://www.mdpi.com/article/10.3390/brainsci16101140/s1, Table S1. School distribution in the principal and full source samples; Table S2. School fixed effects with HC3 uncertainty estimates; Table S3. HC3 sensitivity analyses retaining all source records; Table S4. Exploratory conventional school clustered inference; and Table S5. Summary of leave-one-school-out sensitivity analyses.
