# Supplementary material for: Current Video Gaming Is Associated with Visual–Psychomotor Efficiency and Visuomotor Coordination in Adolescents
Source: Brain Sci. 2026 Oct 21;16(10):1140. doi: 10.3390/brainsci16101140 (PMC13702617; doi:10.3390/brainsci16101140)
Supplement: Supplementary file 1 [file brainsci-16-01140-s001.zip › brainsci-4622213-supplementary.pdf]

Supplementary Material

Current Video Gaming Is Associated with Visual–Psychomotor Efficiency and Visuomotor Coordination in Adolescents

Grzegorz Załęski[1], Krzysztof Horoszkiewicz[1] and Marta Kopańska[2],*

These tables accompany the secondary cross-sectional reanalysis. They report school and sample sensitivity analyses. All comparisons concern current versus non-current gaming; they are not contrasts of lifetime gaming exposure with no exposure. None constitutes an independent replication.

Time effects are adjusted percentage differences in geometric means. Other effects are point differences. q denotes a Benjamini–Hochberg-adjusted p value within the family specified in each table; confidence intervals are pointwise. Models and inferential limitations are described in Section 2.5 of the manuscript.

Table S1

School distribution in the principal and full source samples

School	Current<br>$N = 120$	Non-current<br>$N = 120$	Total<br>$N = 120$	Total<br>$N = 121$
S1	19	1	20	20
S2	2	4	6	6
S3	17	7	24	25
S4	21	5	26	26
S5	5	3	8	8
S6	28	8	36	36

Note. Schools are coded S1–S6. Membership was reconstructed retrospectively. The $N = 121$ source sample additionally includes one non-current boy from S3. Girls came from five schools; no current female players were represented in S5.

Table S2

School fixed effects with HC3 uncertainty estimates

**Sample**	**Outcome**	**Adjusted effect [95% CI]**	***p / q***
Full sample	PiM	+8.05 [+2.53, +13.57]	.005 / .023
Full sample	Number Localization time	-9.5% [-16.3%, -2.1%]	.013 / .033
Full sample	Line Test time	-9.0% [-17.8%, +0.8%]	.071 / .112
Full sample	Line Test accuracy	+0.41 [-0.63, +1.44]	.438 / .438
Full sample	CCTT-2 time	-11.8% [-23.7%, +2.0%]	.089 / .112
Girls	PiM	+9.21 [+3.39, +15.04]	.003 / .013
Girls	Number Localization time	-10.0% [-17.9%, -1.4%]	.025 / .062
Girls	Line Test time	-10.2% [-20.0%, +0.8%]	.068 / .085
Girls	Line Test accuracy	+0.58 [-0.59, +1.74]	.327 / .327
Girls	CCTT-2 time	-15.0% [-28.0%, +0.4%]	.056 / .085

Note. Principal sample $N = 120$; girls $n = 60$. Full-sample models include age, sex, and five school indicators; female-only models include age and four school indicators. Negative percentages indicate shorter geometric mean times; PiM and Line Test accuracy effects are point differences. q is corrected across five outcomes separately within each sample specification. CIs are pointwise. HC3 does not account for within-school dependence.

Table S3

HC3 sensitivity analyses retaining all source records

**Adjustment**	**Outcome**	**Adjusted effect [95% CI]**	***p / q***
Age and sex	PiM	+7.55 [+2.01, +13.10]	.008 / .020
Age and sex	Number Localization time	-9.5% [-15.7%, -2.9%]	.006 / .020
Age and sex	Line Test time	-11.8% [-20.5%, -2.1%]	.018 / .030
Age and sex	Line Test accuracy	+0.26 [-0.67, +1.18]	.584 / .584
Age and sex	CCTT-2 time	-15.8% [-30.7%, +2.4%]	.085 / .106
Age sex and school	PiM	+8.65 [+3.32, +13.97]	.002 / .008
Age sex and school	Number Localization time	-9.4% [-15.8%, -2.5%]	.009 / .023
Age sex and school	Line Test time	-10.3% [-19.1%, -0.7%]	.037 / .061
Age sex and school	Line Test accuracy	+0.40 [-0.57, +1.37]	.416 / .416
Age sex and school	CCTT-2 time	-17.5% [-32.1%, +0.3%]	.053 / .066

Note. $N = 121$. All source records are retained, including the record excluded from principal analyses for internally inconsistent simple-coordination measurements. Inclusion is a sensitivity check, not validation of that record. School-adjusted models include five school indicators. q is corrected across five outcomes separately within each specification. Time effects are percentages; other effects are point differences; CIs are pointwise.

Table S4

Exploratory conventional school clustered inference

**Sample**	**Outcome**	**Adjusted effect [95% CI]**	***p* / *q***
N = 120 full sample	PiM	+8.05 [+4.58, +11.52]	.002 / .008
N = 120 full sample	Number Localization time	-9.5% [-17.8%, -0.3%]	.046 / .077
N = 120 full sample	Line Test time	-9.0% [-13.0%, -4.8%]	.003 / .008
N = 120 full sample	Line Test accuracy	+0.41 [-0.08, +0.90]	.087 / .108
N = 120 full sample	CCTT-2 time	-11.8% [-33.7%, +17.4%]	.311 / .311
Girls n = 60	PiM	+9.21 [+5.43, +13.00]	.003 / .010
Girls n = 60	Number Localization time	-10.0% [-20.3%, +1.6%]	.073 / .091
Girls n = 60	Line Test time	-10.2% [-14.6%, -5.5%]	.004 / .010
Girls n = 60	Line Test accuracy	+0.58 [+0.17, +0.98]	.017 / .028
Girls n = 60	CCTT-2 time	-15.0% [-41.5%, +23.4%]	.293 / .293
N = 121 full sample	PiM	+8.65 [+4.63, +12.66]	.003 / .012
N = 121 full sample	Number Localization time	-9.4% [-17.2%, -0.8%]	.038 / .048
N = 121 full sample	Line Test time	-10.3% [-15.4%, -4.9%]	.005 / .012
N = 121 full sample	Line Test accuracy	+0.40 [-0.04, +0.84]	.068 / .068
N = 121 full sample	CCTT-2 time	-17.5% [-30.6%, -1.9%]	.036 / .048

Note. Models include school fixed effects and the same age or sex covariates as their HC3 counterparts. CR1 multiplier = $G/(G - 1) \times (N - 1)/(N - K)$. t degrees of freedom are five for the full samples and four for girls. Female-only observations are identical in N = 120 and N = 121. q is corrected across five outcomes within each specification. Time effects are percentages; other effects are point differences. With only six or five clusters, these intervals and tests may be unreliable and are exploratory.

Table S5

Summary of leave one school out sensitivity analyses

Sample	Outcome	Adjusted effect range	*q* range	Models with *q* < .05
Full sample	PiM	+7.11 to +8.89	.016–.070	4/6
Full sample	Number Localization time	-11.3% to -5.4%	.012–.460	5/6
Full sample	Line Test time	-10.1% to -8.1%	.113–.220	0/6
Full sample	Line Test accuracy	+0.32 to +0.53	.317–.698	0/6
Full sample	CCTT-2 time	-19.5% to -1.2%	.012–.906	1/6
Girls	PiM	+8.16 to +10.25	.008–.177	4/5
Girls	Number Localization time	-12.3% to -5.8%	.032–.504	1/5
Girls	Line Test time	-11.5% to -8.9%	.085–.369	0/5
Girls	Line Test accuracy	+0.48 to +0.66	.297–.576	0/5
Girls	CCTT-2 time	-23.4% to -2.9%	.017–.782	1/5

Note. These ranges summarize existing school-fixed-effect HC3 models for the principal $N = 120$ sample, omitting each represented school in turn. The full sample contributes six omission models per outcome and girls contribute five. Covariates are age and sex in the full sample and age among girls. *q* is corrected across five outcomes separately within each omission model. The count of models below the threshold is descriptive and is not a further hypothesis test. Effect ranges and *q* ranges need not attain their endpoints in the same omission model. Positive count effects and negative time effects favour current players.
